# Development of Land-Use/Land-Cover Maps Using Landsat-8 and MODIS Data, and Their Integration for Hydro-Ecological Applications

**DOI:** 10.3390/s19224891

**Published:** 2019-11-09

**Authors:** Sadia Afrin, Anil Gupta, Babak Farjad, M. Razu Ahmed, Gopal Achari, Quazi K. Hassan

**Affiliations:** 1 Department of Geomatics Engineering, University of Calgary, 2500 University Drive NW, Calgary, AB T2N 1N4, Canada; sadia.afrin@ucalgary.ca (S.A.); anil.gupta@gov.ab.ca (A.G.); mohammad.ahmed2@ucalgary.ca (M.R.A.); 2 Environmental Monitoring and Science Division, Alberta Environment and Parks, Calgary, AB T2E 7L7, Canada; 3 Department of Civil Engineering, University of Calgary, 2500 University Drive NW, Calgary, AB T2N 1N4, Canada; gachari@ucalgary.ca

**Keywords:** Land use and land cover, hydrology, ecology, wetland, Athabasca River watershed, ISODATA clustering, post-classification modification

## Abstract

The Athabasca River watershed plays a dominant role in both the economy and the environment in Alberta, Canada. Natural and anthropogenic factors rapidly changed the landscape of the watershed in recent decades. The dynamic of such changes in the landscape characteristics of the watershed calls for a comprehensive and up-to-date land-use and land-cover (LULC) map, which could serve different user-groups and purposes. The aim of the study herein was to delineate a 2016 LULC map of the Athabasca River watershed using Landsat-8 Operational Land Imager (OLI) images, Moderate Resolution Imaging Spectroradiometer (MODIS)-derived enhanced vegetation index (EVI) images, and other ancillary data. In order to achieve this, firstly, a preliminary LULC map was developed through applying the iterative self-organizing data analysis (ISODATA) clustering technique on 24 scenes of Landsat-8 OLI. Secondly, a Terra MODIS-derived 250-m 16-day composite of 30 EVI images over the growing season was employed to enhance the vegetation classes. Thirdly, several geospatial ancillary datasets were used in the post-classification improvement processes to generate a final 2016 LULC map of the study area, exhibiting 14 LULC classes. Fourthly, an accuracy assessment was carried out to ensure the reliability of the generated final LULC classes. The results, with an overall accuracy and Cohen’s kappa of 74.95% and 68.34%, respectively, showed that coniferous forest (47.30%), deciduous forest (16.76%), mixed forest (6.65%), agriculture (6.37%), water (6.10%), and developed land (3.78%) were the major LULC classes of the watershed. Fifthly, to support the data needs of scientists across various disciplines, data fusion techniques into the LULC map were performed using the Alberta merged wetland inventory 2017 data. The results generated two useful maps applicable for hydro-ecological applications. Such maps depicted two specific categories including different types of burned (approximately 6%) and wetland (approximately 30%) classes. In fact, these maps could serve as important decision support tools for policy-makers and local regulatory authorities in the sustainable management of the Athabasca River watershed.

## 1. Introduction

Land cover refers to the physical cover of the earth’s surface, and land use refers to the functional use of land by humans [1,2]. Land-use and land-cover (LULC) maps are used as powerful tools for tackling environmental problems, facilitating planning activities, and managing natural resources such as water resources [3]. The Athabasca River watershed, located in the northern part of the province of Alberta in Canada, plays a dominant role in the province’s economy since the emergence of the oil and gas industry in 1967. Moreover, the watershed comprises boreal forest, which strongly influences the environmental biodiversity of the province and provides livelihood to Indigenous people living there. The watershed changed rapidly in the last few decades due to urbanization and industrial activities including coal and oil sand mining, natural gas extraction, agricultural expansion, forest degradation, and pulp and paper production [4,5,6]. In addition to these man-made activities, natural hazards such as wildfire also altered the landscape of the watershed [7]. Therefore, developing a comprehensive and up-to-date LULC map is imperative to better understanding the complex and dynamic characteristics of the Athabasca River watershed.

Both land use and land cover can greatly impact hydro-ecological processes of the watershed including ecosystem productivity, evapotranspiration, soil infiltration, and runoff [8,9,10,11,12]. Some studies [11,13] showed that agricultural practices, urbanization, deforestation, and industrialization are major drivers affecting water quality and quantity. For example, the excessive sedimentation from agricultural practices results in non-point pollutions from diffuse sources to the water quality [14]. In addition, urbanization and impervious surfaces impede precipitation infiltration and increase stream flow [10,12]. Deforestation through wildfire, logging, or constructions also affects evapotranspiration and soil infiltration [13]. In Alberta, most oil and gas activities are located near the lower Athabasca River. Therefore, the assessments of these activities are of critical importance for ecologists and hydrologists due to the large scale of operations. Oil and gas activities also interrupt the natural hydrological characteristics of watersheds by diverting stream flows and altering natural drainage patterns during site construction and extraction of groundwater or surface water from the nearby waterbodies during operations [15]. According to the provincial water allocation in 2012, the oil and gas sector is allowed to use 10% (2% for conventional oil and gas and 8% for oil sand) of Alberta’s total surface water allocation [16]. Approximately 60% of the water usage in the oil sand industry is extracted from the Athabasca river [17]. In 2018, 2.6 barrels of non-saline water were needed to produce one barrel of oil equivalent (BOE) [17]. Such non-saline water was extracted from surface runoff (mainly from Athabasca River) and groundwater within the mining footprint. Furthermore, chemical emissions from the oil and gas industries in the Athabasca River watershed may create stress on the aquatic environment [18].

In delineating the spatiotemporal dynamics of LULC, satellite-based remote sensing data can easily provide comprehensive information about an area. The use of satellite data became popular in the 1980s with the increasing and consistent availability of satellite images to the public. LULC studies usually applied satellite data with moderate (250 m) to high (5 m) spatial resolution [19,20], depending on the detail requirements and available resources. Satellite data with coarser spatial resolution require less computation time but provide less detailed information and fail to detect any landscape changes that are lower than the satellite sensor’s spatial resolution on the ground [21,22]. In contrast, satellite data with higher spatial resolution provide finer details on LULC information. Nevertheless, high-resolution data require rigorous computations and longer processing times [23]. Their usages are limited to smaller study areas due to the lack of historical time-series data and the higher cost requirements in image acquisition. In order to overcome such issues, Moderate Resolution Imaging Spectroradiometer (MODIS; spatial resolution 250 m) and Landsat data (spatial resolution 30 m) are widely used in LULC studies. Although both MODIS and Landsat data archives offer open access to spectrally and geometrically corrected high-quality multispectral band information, MODIS data should be cautiously used in local-level LULC studies due to its relatively coarser spatial resolution [24].

Landsat is the most used satellite in LULC studies. Since the opening of Landsat’s archive in 2008, the use of Landsat data significantly increased because of the free access to its 40 years of earth observation data [25]. Many countries generated nation-wide LULC maps primarily based on Landsat data; examples are (i) the Coordination of Information on the Environment (CORINE) Program of the 39 European countries [26], (ii) the National Land Cover Database (NLCD) of the United State of America [1], and (iii) the national Land Cover Map 2015 (LCM2015) of Great Britain [27]. In Canada, Landsat data are also popular in LULC mapping at all scales. At the national scale, the most recent national land-cover dataset was generated under the North American Land Change Monitoring System (NALCMS) collaboration using Landsat 5 and Landsat 7 satellite imagery [28]. Two other popular national-level maps, the Earth Observation for Sustainable Development of Forests (EOSD) [29] land-cover map for forested areas and the Agriculture and Agri-Food Canada (AAFC) [30] land-use map for agricultural areas, were also delineated based on Landsat data. At the regional level in 2014, the Alberta Biodiversity Monitoring Institute (ABMI) produced a series of two land-cover maps circa 2000 and 2010 for the entire province of Alberta using Landsat data [31]. These maps were considered to be the most accurate land-cover maps available for Alberta with an overall accuracy of 75.92% [31]. However, those maps had two specific issues, i.e., overestimation of shrub class and no existence of wetland and wildfire classes, which would complicate their usability for the Athabasca River watershed. Therefore, an updated version of LULC maps is required, as two major wildfires and rapid urbanization further altered the LULC of the watershed between 2010 and 2016.

In general, in order to generate a Landsat-based LULC map over a large geographical area like the Athabasca River watershed for a given year, it would be challenging to obtain good-quality Landsat images as they would often be impacted by cloudy conditions [25]. To overcome these limitations, most studies replaced the contaminated scene with an alternative clear scene acquired on a later date. However, finding an immediate replacement is sometimes difficult due to Landsat’s long (16 days) temporal resolution. In such cases, an alternate month or an alternate year with a clear scene was used as an image replacement. This might have caused spatial inconsistencies with the possibility of missing some important landscape characteristics. An earlier study that encountered similar problems suggested including multidate Landsat imagery for vegetation classification [19]. However, this suggestion is not feasible for the Athabasca River watershed, as the presence of the Rocky Mountains in the watershed, as well as wildfires and weather conditions, would frequently create image contaminations resulting from cloud and haze. Hence, the readily available and cloud-free temporal series of MODIS vegetation index products were used as an alternative solution for capturing the spatiotemporal dynamics of vegetation types. Many ecological and forest studies applied the knowledge of plant phenology to identify the vegetation types [32,33]. Using a time series of MODIS-derived vegetation index data, these studies confirmed that the surface reflectance of the boreal forest changes over the growing season due to the phenological characteristics of the deciduous trees. The study herein used the same approach to classify the vegetation coverage of the watershed.

In this context, the study presents a unique, integrated, and cost-effective approach of using Landsat 8 Operational Land Imager (OLI) and MODIS data to generate a comprehensive LULC map of the Athabasca River watershed, which could be used to facilitate local level planning activities, environmental monitoring, and natural resource management of the watershed. Moreover, this study customizes the LULC map to satisfy the specific requirements of the ecologists and hydrologists. The specific objectives of the study area are to (i) delineate a 30-m-spatial-resolution land-use and land-cover maps of the study area in 2016 using Landsat 8 OLI and a time series of MODIS vegetation index images, and (ii) generate two user-friendly maps incorporating wetland information into the LULC map for hydro-ecological applications.

## 2. Materials and Methods

### 2.1. The Study Area

**Figure 1:**
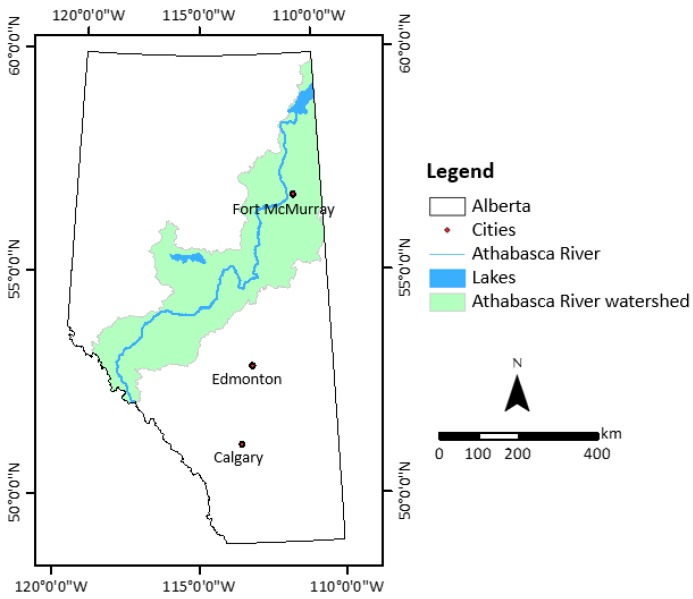
Location of the Athabasca River watershed within the Canadian Province of Alberta.

The Athabasca River watershed is located between the two adjacent prairie provinces of Alberta and Saskatchewan; however, the study area comprises the watershed area (about 90% of the entire watershed) located within Alberta. The total land mass of the study area is approximately 151,220 km^2^, which is almost one-quarter of the total provincial land (Figure 1) [34]. The area includes boreal forests, the Canadian shield, foothills, the Rocky Mountains, and a small portion of the Parkland Natural Region of Alberta [35]. In the study area, boreal forests and mixed-wood forests cover a major portion of the watershed, whereas concentrated human settlements are confined to a few urbanized areas like Fort McMurray, Slave Lake, Hinton, Whitecourt, Edson, Jasper, and Athabasca.

The study area can be divided into three segments based on its very distinct landscape characteristics. The western side of the study segment is a rocky area, which includes Jasper National Park, a federally protected area which limits landscape changes in the upper part of the watershed. Next, the middle portion of the watershed is dominated by agricultural land use, boreal forests, and mixed-wood central forests. Characterized by ample natural resources, this area plays an important environmental role in maintaining natural biodiversity at local to regional levels. However, abrupt landscape changes often occur in this portion due to agricultural land conversion, natural resource extraction, wildfires, insect infiltration or infection, and forest harvesting/cut blocks. The eastern side is the most dynamic region of the watershed, as it went through intense urbanization through the massive expansion of the oil and gas industry in the last few decades. The oil and gas sector located in this region generated one-third of all provincial economic activity and contributed approximately seven billion Canadian dollars (CAD) to Alberta’s economy in 2015 [36]. Despite this industry’s tremendous economic contribution to the province, environmentalists and policy-makers are deeply concerned about the environmental impacts of these activities. The entire watershed faces the imminent threats of frequent wildfires due to climate change and increased human accessibility to the boreal forest area between 2000 and 2014 [7]. The last two major wildfires (known as the Lesser Slave Lake fire in 2011 and the Fort MacMurray (Horse River) fire in 2016) altered the forest coverage of the watershed. Together, they affected more than 100,000 people and caused more than 9.5 billion CAD in economic losses [37].

### 2.2. Data Requirements

**Figure 2:**
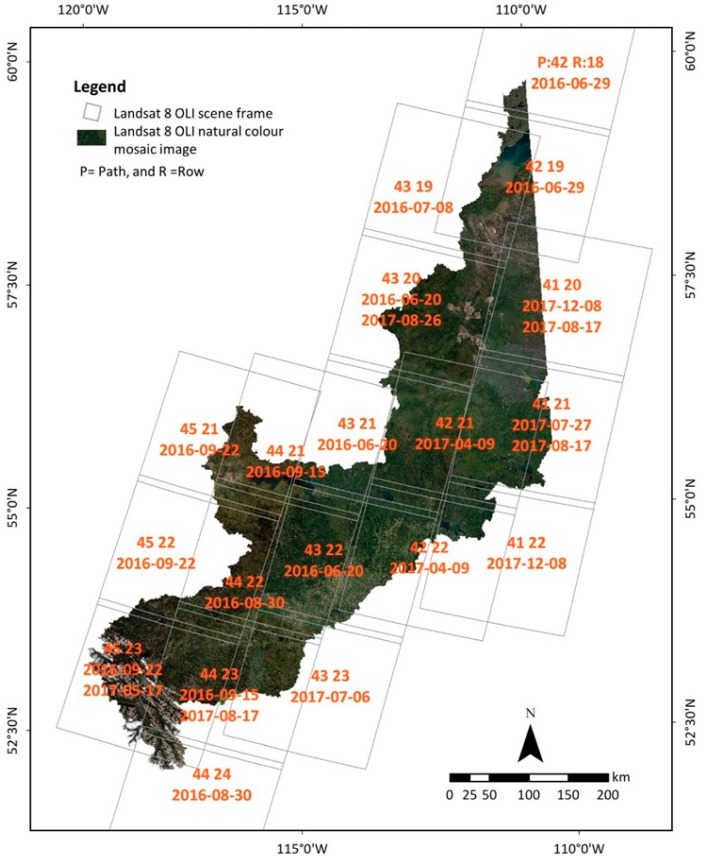
Landsat-8 Operational Land Imager (OLI) mosaic image of the study area displaying frames of the 24 scenes with their path and row and their acquisition dates.

In this study, the LULC maps were developed based on three key datasets: (i) Landsat-8 OLI satellite imagery received from the United States Geological Survey (USGS); (ii) MODIS/Terra 16-day composite enhanced vegetation index (EVI) product (MOD13Q1) acquired from the National Aeronautics and Space Administration (NASA), and (iii) ancillary data collected from the Government of Alberta (https://geodiscover.alberta.ca/geoportal/) and the Alberta Biodiversity Monitoring Institute (http://www.abmi.ca/home/data-analytics). The LULC of the study area classified primarily based on 30-m-spatial-resolution Landsat-8 OLI multispectral images by using the seven visible and near-infrared (VNIR) bands covering wavelengths from 0.43–2.29 µm. The seven VNIR bands of Landsat-8 OLI were coastal aerosol (C, 0.43–0.45 µm), blue (B, 0.45–0.51 µm), green (G, 0.53–0.59 µm), red (R, 0.64–0.67 µm), near-infrared (NIR, 0.85–0.88 µm), shortwave infrared 1 (SWIR 1, 1.57–1.65 µm), and shortwave infrared 2 (SWIR 2, 2.11–2.29 µm). Upon consulting the Landsat-8 image database, it was revealed that a sufficient number of cloud-free images was unavailable during the growing season (May to September) in 2016 to cover the entire study area. Thus, the images acquired in 2017′s growing season were also considered. Finally, a total of 24 Landsat-8 images were collected during the 2016 and 2017 growing seasons, and their path and row, along with their acquisition dates, are shown in Figure 2. Note that it is common practice in Canada (both at the national and provincial levels) to use multi-year Landsat data to cover a large area, which includes EOSD’s land-cover map for forested areas [29], AAFC’s land-use map for agricultural areas [30], and ABMI’s two land-cover maps circa 2000 and 2010 [31]. Consequently, the usage of 2016 and 2017 Landsat images to produce an LULC map for circa 2016 would be quite acceptable.

In addition, MODIS/Terra 16-day composite EVI images with 250-m spatial resolution (MOD13Q1) were used over the growing season (day of year (DOY) 129 to DOY 273) of 2016 to generate an accurate vegetation coverage of the watershed. The study area required three tiles of MOD13Q1, and each tile had 10 temporal images over the growing season. Therefore, a total of 30 EVI images of MOD13Q1 were used. Furthermore, some ancillary government data and information, such as historical LULC maps (i.e., the land-use map of AAFC 2010 and the wall-to-wall land-cover inventory (LCI) of ABMI 2010) were used for qualitative evaluation of the generated preliminary LULC maps. In addition, the historical wildfire perimeter map of 2017 and the Alberta Merged Wetland Inventory of 2017, from the Government of Alberta’s online resources, were used to enhance the final LULC maps, making them more informative. Additionally, the road and rail networks, and the power and transmission lines of the study area were extracted from the 2016 Human Footprint Inventory (HFI, Version 1) dataset collected by the Alberta Biodiversity Monitoring Institute. The authors integrated these extracted layers into the LULC maps to enhance the spatial accuracy of the linear features. In order to validate the final LULC maps, secondary data sources were used, including the historical imageries available in Google Earth Pro, the SPOT-6 (Satellite pour l’Observation de la Terre) imageries of 2016 collected from the Alberta Environment and Parks (AEP), and the 3 × 7-km photo plot land cover (PPLC) 2016 dataset received from the ABMI.

## 3. Methods

**Figure 3:**
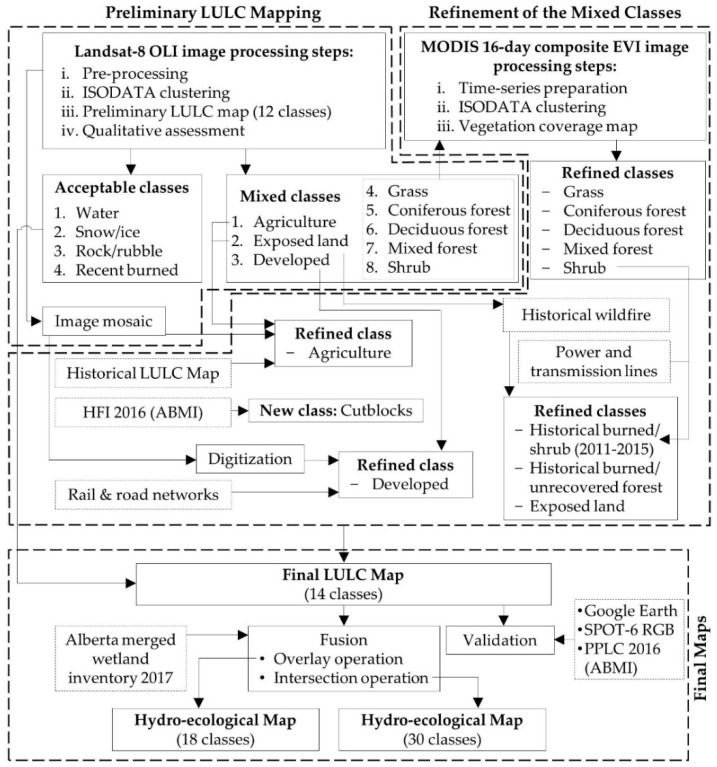
A schematic diagram of the methods followed in this study demonstrating the satellite image-based delineation of the land-use and land-cover (LULC) map and its enhancement for use in hydro-ecological applications for the Athabasca River watershed.

The schematic diagram of the methods used in the study is shown in Figure 3. Three major components were performed: (i) generating a preliminary LULC map of 2016 at 30-m spatial resolution based on Landsat-8 OLI data, (ii) refining the mixed classes using MODIS and other ancillary data to derive a final LULC map and its validation, and (iii) preparing the hydro-ecological maps. Each component is discussed in the sub-sections below.

### 3.1. Generating the Preliminary LULC Map

After collecting Landsat-8 OLI multispectral imagery, the following three pre-processing steps were carried out on 24 Landsat image scenes individually: (i) layer-stacking seven multispectral bands of each Landsat-8 OLI image, (ii) re-projecting each image to NAD 83 UTM 10 AEP Forest, the projection used by the Government of Alberta, and (iii) sub-setting the images to the extent of the study area. Next, the iterative self-organizing data analysis (ISODATA) clustering technique (an unsupervised classification) was performed on each layer-stacked image that initially produced 50 classes with a convergence threshold of 0.995 in 500 iterations. The clusters were further regrouped into 12 LULC classes based on the similarity in patterns of cluster-specific spectral signatures. By combining the 24 classified Landsat-8 images, a preliminary LULC map of the study area was generated with the following 12 LULC classes: water, snow/ice, rock/rubble, recent burned, agriculture, exposed land, developed, grass, coniferous forest, deciduous forest, mixed forest, and shrub. Thereafter, the preliminary LULC map was qualitatively assessed through a visual comparison with historical LULC maps (i.e., the land-use map of AAFC 2010 and the wall-to-wall land-cover inventory (LCI) 2010 of ABMI).

### 3.2. Rectifying the Mixed Classes

Based on the qualitative assessment of the preliminary LULC map, we determined two sets of classes, i.e., acceptable and mixed. The acceptable class set included water, snow/ice, rock/rubble, and recent burned classes, and the mixed classes were agriculture, exposed land, developed, grass, coniferous forest, deciduous forest, mixed forest, and shrub. It was observed that developed, exposed land, agriculture (bare types), and vegetation classes (i.e., grass, coniferous forest, deciduous forest, mixed forest, and shrub) were misclassified in several locations. For example, “exposed land” and “developed” classes were mixed and misclassified as each other. Additionally, bare agriculture areas were not well separated from some of the exposed lands and, thus, missed some current fallow agriculture lands in the “agriculture” class. Furthermore, vegetation classes, including the forest classes, i.e., coniferous, deciduous, and mixed, were also mixed and misclassified as each other in several locations. To rectify all these misclassified classes, some enhancements of the preliminary LULC map were performed by using MODIS-derived EVI images (MOD13Q1) and ancillary data, as described below.

#### 3.2.1. Rectifying Vegetation Classes

For rectifying the vegetation classes, three gridded tiles of MOD13Q 1 EVI images were used to cover the entire study area. A total of 30 EVI images were acquired for preparing the time series of vegetation growing period (i.e., DOY 129 to DOY 273 of 2016), where each tile extent corresponded to 10 EVI images. Next, three time-series images corresponding to three tiles were prepared by layer-stacking of 10 EVI images for each tile. Such an operation allowed us to observe the temporal variation of vegetation in the EVI images (time series) over the growing season of 2016. Here, the intention was to extract those pixels from each of the EVI composite images that were spatially intersected with the misclassified pixels of the five vegetation classes (i.e., grass, coniferous forest, deciduous forest, mixed forest, and shrub) in the preliminary Landsat-derived LULC classes. Thereafter, ISODATA classification was applied to each set of time-series EVI images to generate 20 initial classes with a convergence threshold of 0.995 and 250 iterations. Subsequently, those 20 classes were regrouped into the intended five vegetation classes based on the patterns of cluster-specific spatiotemporal profiles. Finally, the three classified time-series images were spatially combined to prepare a vegetation coverage map of the entire study area. These five vegetation classes replaced the vegetation in the final LULC map. At this stage, this vegetation coverage map was resampled to 30 m to match the resolution of the Landsat-derived LULC map.

#### 3.2.2. Rectifying Developed, Exposed Land, and Agriculture Classes

In order to rectify the developed, exposed land, and agriculture (bare) classes, several methods were implemented with the help of several ancillary data. A mosaic image was prepared initially for the study area from the cloud-free areas of 24 pre-processed Landsat-8 OLI images. For developed areas, such as urban lands, boundaries were digitized on the mosaic image, and considered as the “developed” class. The digitization approach was chosen since the urban areas were relatively small in comparison to the entire study area, and were confined to some specific known locations. Other smaller developed areas, such as mining sites, were also digitized based on visual scanning of the mosaic image with different band combinations, and using ABMI’s HFI 2016. In addition, rail and road networks were also considered as the “developed” class, which were derived from the same ABMI HFI 2016 data. At this stage, the “developed” class of the preliminary LULC map was enhanced by replacing it with all these new and revised developed areas.

Furthermore, the enhancement of the “agriculture” class was performed by identifying the class in historical LULC maps (i.e., ABMI’s LCI 2010 and AAFC’s land-use map 2010) and relating it to the exposed lands with parcel-like patterns in the Landsat-8 mosaic image. Zones were prepared based on the patterns of agriculture parcel in the mosaic image and their presence as agriculture in the historical LULC maps. In the case of any “exposed land” class of the preliminary LULC map being located within any agricultural zone, it was considered as the “agriculture” class. These types of bare or exposed lands were essentially the current fallow but agriculture area during the image acquisition dates and they were, thus, considered under the “agriculture” class. However, the exposed lands that did not follow any patterns of agriculture parcel (i.e., outside the agricultural zones) were left as the “exposed land” class.

The abovementioned criteria in the previous paragraph that would be applicable as agricultural practices are mainly restricted to some agricultural zones because of the topographical and climatic conditions. Also, our study area is located in the northern part of Alberta, where cold weather impedes agricultural practices. Moreover, most of the study area is covered by boreal forest, and human settlements are extremely limited in the northern part, especially toward the north from the Fort McMurray urban service area. Finally, the exposed lands that follow regular patterns in the oil sand region are mainly clear cuts for mining activities/forest management. Consequently, the assumption was that the agricultural areas did not expand and, if they did, the change was insignificant.

### 3.3. Generating the Final LULC Map by Integrating the Rectifications

During the process of integrating the new and revised vegetation, developed, exposed land, and agriculture classes into the preliminary LULC map, a priority rule was applied. For example, the highest priority was given to the “developed” class, which included road and rail networks, so that it could keep continuity across the watershed. After the integrations, it was observed that the exposed land became a dominant LULC category in the study area, which did not comply with the available historical maps. After careful observation, it was revealed that the study area underwent several wildfires in the past, and those burned locations were the main cause of the issue. To resolve this, the Government of Albert’s historical wildfire perimeter database 2017 was used. In this case, any exposed land within the wildfire perimeter during the recent past (2011–2015) was considered as the shrub category and, thus, the “shrub” class in the final LULC map was renamed as the “historical burned/shrub” class. Additionally, in the case of any “exposed land” class of the preliminary LULC map being located within the perimeter of wildfires which occurred before 2011, it was named “historical burned/unrecovered forest”, which was a newly added class in the final LULC map.

A final post-classification refinement was conducted by adding some additional information from ABMI’s Human Footprint Inventory database of 2016 [38] to remain consistent with the definitions and classes available in ABMI’s historical LULC maps. For example, cutblocks between 2011 and 2016 were systematically included as the “cutblocks” class in the final LULC map. Also, power and transmission lines were included in the final LULC map as the “historical burned/shrub” class only if they intervened with vegetation classes. After all the above integrations and post-classification modifications, the final LULC map of the study area with a total of 14 classes (including the “historical burned/shrub” and “historical burned/unrecovered forest” classes, in addition to the preliminary 12 classes) was spatially filtered with a 3 × 3 window to enhance and smoothen it.

### 3.4. Validating the Final LULC Map

To ensure the credibility and reliability of the final LULC map, an accuracy assessment was performed by calculating the overall accuracy and kappa coefficient using the following expressions (Equations (1) and (2)):

(1) $$\mathrm{Overall}\;\mathrm{Accuracy}=\frac{\sum \mathrm{All}\;\mathrm{diagonal}\;\mathrm{values}\;\mathrm{in}\;\mathrm{the}\;\mathrm{confusion}\;\mathrm{matrix}}{\mathrm{Total}\;\mathrm{number}\;\mathrm{of}\;\mathrm{observation}\;\mathrm{included}\;\mathrm{in}\;\mathrm{the}\;\mathrm{matrix}},$$

(2) $$\hat{K}=\frac{N\sum_{i=1}^{r}X_{ii}-\sum_{i=1}^{r}\left(X_{i+}*X_{+i}\right)}{N^{2}-\sum_{i=1}^{r}\left(X_{i+}*X_{+i}\right)},$$

where $\hat{K}$ is the kappa value, *r* is the number of row/column(s) in the confusion (error) matrix, *X_ii_* is the number of observations in row *i* and column *i* (on the major diagonal), *X_i_*_+_ represents the total observations in row *i*, *X*_+*i*_ represents the total observations in column *i*, and *N* is the total number of observations included in the matrix.

For the accuracy assessment, the producer’s and user’s accuracies of the LULC classes were assessed. In the process, 12 out of 14 LULC classes were considered, which were primarily determined based on their spectral responses. The remaining two classes (i.e., “historical burned/unrecovered forest” and “cutblocks”) were excluded from the assessment, because they were directly derived from the polygons of the ancillary geospatial data. To accomplish the process, another set of ancillary reference data was used, which was not applied during the generation processes of the final LULC map, whereby reference polygons of agriculture, grass, and snow/ice classes were derived from Google Earth Pro’s historical images, those of developed and recent burned classes were derived from a SPOT-6 red/green/blue (RGB) mosaic image with a spatial resolution of 1.5 m, and the remaining classes were derived from ABMI’s 3 × 7-km photo plot land-cover data 2016. The ABMI dataset included LULC classes collected from 1656 permanent sample sites of a 3 × 7-km area evenly spaced on a 20-km grid across Alberta [39]. A total of 252 photo plots were located within the study area, and those were used. A stratified random sampling method was followed to generate the reference polygons from the reference dataset for each of the 12 LULC classes. Note that no direct ground reference data were used in the assessment process, because collecting ground data from field surveys was out of the scope of this study due to insufficient resources.

### 3.5. Preparing the Hydro-Ecological Maps

Two different types of hydro-ecological map of the study area were prepared to support the data needs of scientists across various disciplines by incorporating the Alberta Merged Wetland Inventory (AMWI) 2017 dataset [40] (produced by Government of Alberta) into the final LULC map. The AMWI dataset of 2017 included five major classes of the Canadian Wetland Classification System (CWCS), i.e., bog, fen, marsh, swamp, and open water, for the entire province of Alberta. The minimum map unit of the inventory was 0.0009 km^2^ with a horizontal accuracy of 30 m. The overall accuracies of the AMWI maps located in the Athabasca River watershed area were reported to vary from 92.4% to 97.17%.

To generate two different hydro-ecological maps, four different types of wetland class (i.e., bog, fen, marsh, and swamp) of the AMWI dataset were fused with the final LULC map (generated in Section 3.1) using two different approaches. Note that the open water class from the AMWI dataset was not used in this process. This was due to the fact that the AMWI database (released in March 2017) used Landsat imagery available over the period 1998 to 2015 for wetland classification [40]. On the contrary, this study used Landsat imagery from 2016 and 2017; thus, the open water class derived from these images was used. For the first hydro-ecological map, the four CWCS wetland classes were directly superimposed (overlaid) on the final LULC map, where other classes of the final LULC map within wetland polygons were eliminated. Therefore, the first hydro-ecological map demonstrated a total of 18 classes. In contrast, the second hydro-ecological map was generated with more elaborate and detailed information about the land-cover types with wetlands and, therefore, it included a total of 30 classes. In this map, instead of eliminating the final LULC classes in the wetland polygons, each CWCS wetland class was further subdivided into four classes based on their intersections with coniferous forest, deciduous forest, mixed forest, and other classes of the final LULC map. However, for both types of hydro-ecological map, developed and recent burned classes were considered as non-changeable from the final LULC map, even though some of those overlapped or intersected with wetland classes in a few locations.

## 4. Results and Discussion

### 4.1. Final LULC Map

Common approaches using Landsat-8 OLI multispectral images were followed (Section 3.1) to delineate the preliminary LULC map of the study area. However, the results of the qualitative and quantitative evaluations of the map detected an unsatisfactory LULC classification with three shortcomings. Firstly, fallow agricultural lands (i.e., agriculture class) were misclassified as exposed land because of having similar spectral signatures. Secondly, developed and exposed land classes were mixed up in some cases due to their close spectral signature patterns. Thirdly and most critically, vegetation classes were frequently misclassified because of their spectral variation throughout the growing season. It was also found that some vegetation patches in the boreal forest regions, especially in deciduous forest areas, lacked continuity among adjacent images that were acquired in different phenological periods, i.e., at different times of the year, or even in different years. This seasonal discrepancy in vegetation classification mainly occurred due to differences in plant understory conditions, bud break, chlorophyll absorption rates, water moisture levels, and leaf biomass levels [41]. Such findings resonated with the findings of a Castilla et al. [19] study, which stated that the use of single-dated Landsat imagery alone cannot accurately generate an LULC map of a large watershed with a highly heterogeneous landscape, such as the study area herein. However, multi-dated and multi-scene Landsat-8 images within a two-year (2016–2017) time frame were used herein for deriving the cloud-free data coverage of the entire extent of the study area.

**Figure 4:**
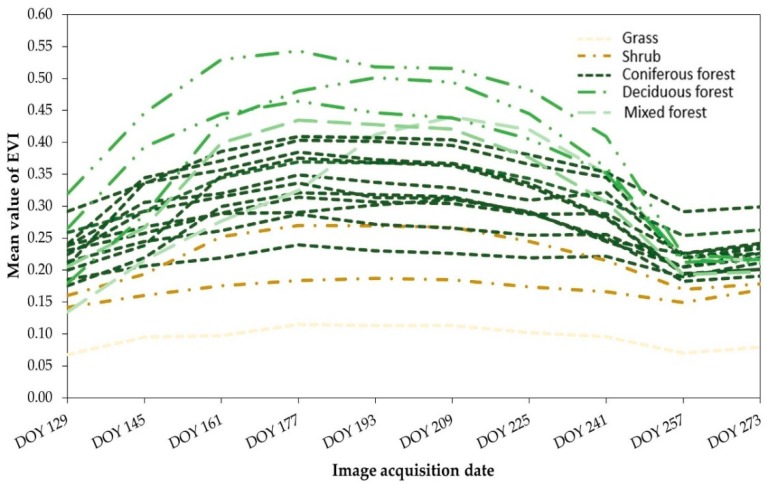
Temporal profiles of the Moderate Resolution Imaging Spectroradiometer (MODIS)/Terra 16-day composite (MOD13Q1) enhanced vegetation index (EVI) images for a tile area (h12v03) indicating phenological changes in vegetation classes (20 lines for the 20 initial classes that were later regrouped into five classes) over the growing season of 2016.

As more than 80% of the total study area is covered by different types of vegetation, modifying the inaccuracies in the vegetation classes in the preliminary LULC map was deemed necessary. As such, a vegetation coverage map of the study area was prepared using a temporal series (time series) of MODIS/Terra 16-day composite (MOD13Q1) EVI images with a 250-m spatial resolution over the growing season of 2016. Figure 4 shows the temporal profiles of the vegetation classes in the study area derived from the time series of EVI images from DOY 129 to DOY 273 (May to September) of 2016. Based on the phenological characteristics of the boreal forest regions of Alberta and temporal profiles of mean EVI values, five vegetation classes (i.e., grass, shrub, coniferous forest, deciduous forest, and mixed forest; see Figure 4) were identified in the vegetation coverage map. These vegetation classes eventually contributed to finalizing the LULC map of the study area.

**Figure 5:**
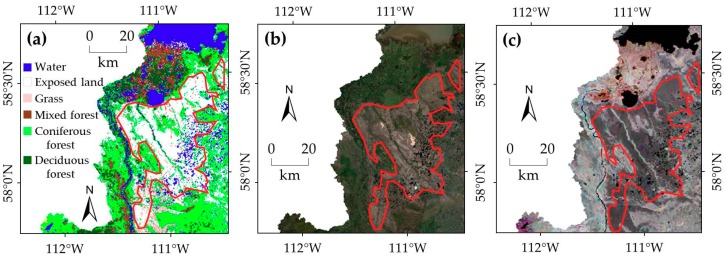
A large area of exposed land (roughly bounded by red line) in the northeastern part of the study area in (**a**) the preliminary LULC map; (**b**) the Landsat-8 OLI natural-color mosaic image; and (**c**) the MODIS/Terra 16-day composite EVI mosaic image between day of year (DOY) 129 and DOY 273.

The limitations of the preliminary LULC map were overcome by executing a series of post-classification refinements based on ancillary data. Indeed, numerous large-area LULC classification studies depended on extensive post-classification refinements or enhancements as identified by Franklin and Wulder [42]. However, the success of these refinements depends on enough and reliable ancillary data, which were available for the study area. Adding up-to-date cutblock, rail and road network, powerline, and transmission line information to the final LULC map made it more comprehensive. Moreover, the use of other ancillary data in the post-classification refinements increased the thematic accuracy of the final map. For instance, the preliminary LULC map also demonstrated a large chunk of exposed land in the northeast part of the watershed (Figure 5a). This finding complied with the Landsat mosaic image and MODIS EVI images used in this study (Figure 5b,c), yet it contradicted the historical LULC maps. These maps identified the exposed land chunk as coniferous-dominated forested area. This issue was resolved by applying the decision rules described in Section 3.2.

**Figure 6:**
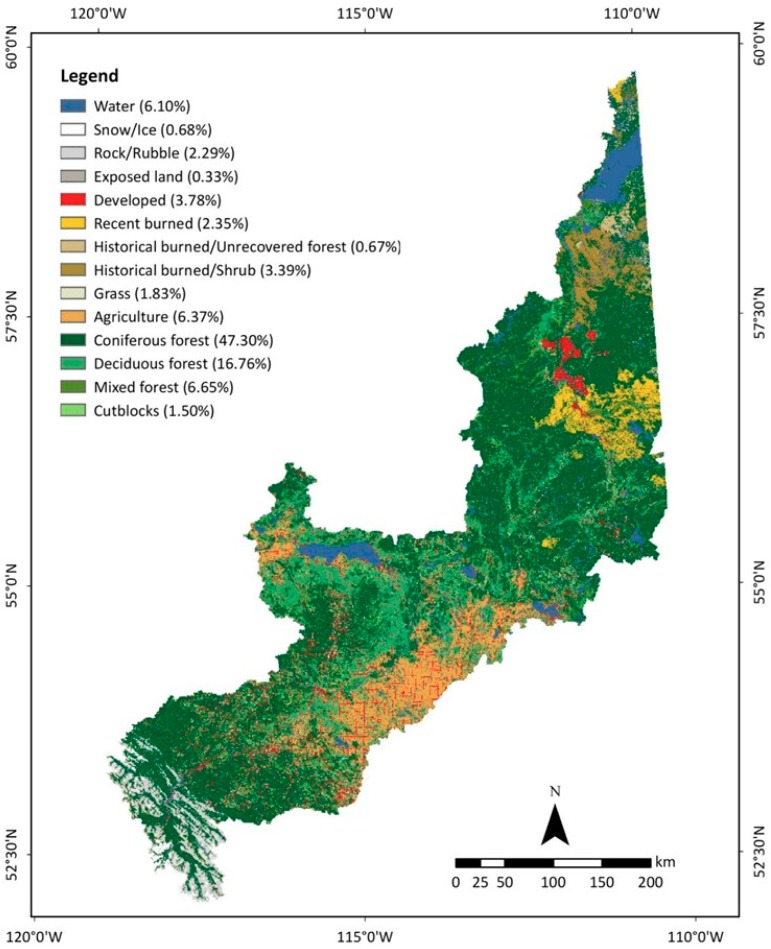
The final LULC map of the study area circa 2016 consisting of 14 LULC classes. The color scheme of the North American Land Change Monitoring system (NALCMS, 2005) is followed. The name and percentage of coverage of each LULC class in the study area is printed in the legend.

The final LULC map of circa 2016 provided comprehensive LULC information demonstrating 14 different LULC classes with their percentage of coverage in the study area (see Figure 6). Numerous studies suggested that the comprehensive information on land use and land cover (LULC) is critical for comprehending local–regional–global environmental issues, such as climate change [43,44], water pollution [45,46], air pollution [47], urban expansion [48], forest depletion [4,49], and habitat fragmentation [50]. This study identified that the entire watershed is immensely vegetated with coniferous forest (47.30%), deciduous forest (16.76%), and mixed forest (6.65%). The other dominant category of LULC class was agriculture (6.37%), which was followed by water (6.10%), developed (3.78%), historical burned/shrub (3.39%), and recent burned (2.35%). The final LULC map lacked CWCS wetland classes, an important LULC feature of the study area, due to the class contradiction of spectral signatures with other vegetation classes used in the map, which was resolved by integrating the AWMI 2017 dataset [40] with the final LULC map.

### 4.2. Validation of the Final LULC Map

The final LULC map derived from Landsat-8 OLI was validated based on the following 12 classes: water, snow/ice, rock/rubble, exposed land, developed, recent burned, historical burned/shrub, grass, agriculture, coniferous forest, deciduous forest, and mixed forest. Table 1 shows the class-wise LULC distribution of the reference area, and the total area used in the accuracy assessment. The total reference area was 2060 km^2^, which accounted for 1.37% of the total study area; the reference dataset included at least 1% of the area covered by the respective 12 classes in the final LULC map.

Table 2 presents the confusion matrix resulting from the accuracy assessment of the final LULC map. The overall accuracy of thematic classes of the final LULC map was 74.95%, and Cohen’s kappa was 0.68, as estimated by the reference dataset. Although the overall accuracy was below 80%, it was acceptable for a large watershed with heterogeneous LULC classes. Moreover, the overall accuracy was similar to that of Castilla et al.’s study [19], which computed an overall accuracy of 75.92% for 10 LULC classes in Alberta. Table 2 also demonstrates that the producer’s accuracy of the LULC classes was over 80% except for deciduous forest (54%) and mixed forest (23%). In addition, the user’s accuracy varied from 26% to 98% with low user’s accuracies for mixed forest (26%), exposed land (49%), grass (53%), and historical burned/shrub (59%). It was identified that the lower producer’s and user’s accuracies in these classes were partly due to some inconsistencies in the definitions of reference data and the final LULC data. Moreover, it was acknowledged that defining mixed forest based on its spectral signature is a complicated process, which may have been the source of uncertainty in that class information in the final LULC map. Finally, exposed land and grass demonstrated smaller coverages, i.e., 0.33% and 1.83%, respectively, in the final LULC map. Therefore, merging these tinier coverages of LULC classes may increase the overall accuracy of the final LULC map. In addition, it would be worthwhile to note that integrating machine learning, implementing geographic object-based image analysis (GEOBIA) practices, or fusing more high-spatial-resolution remote sensing data would potentially enhance such accuracies. However, these approaches were beyond the scope of the current study.

### 4.3. Hydro-Ecological Maps

**Figure 7:**
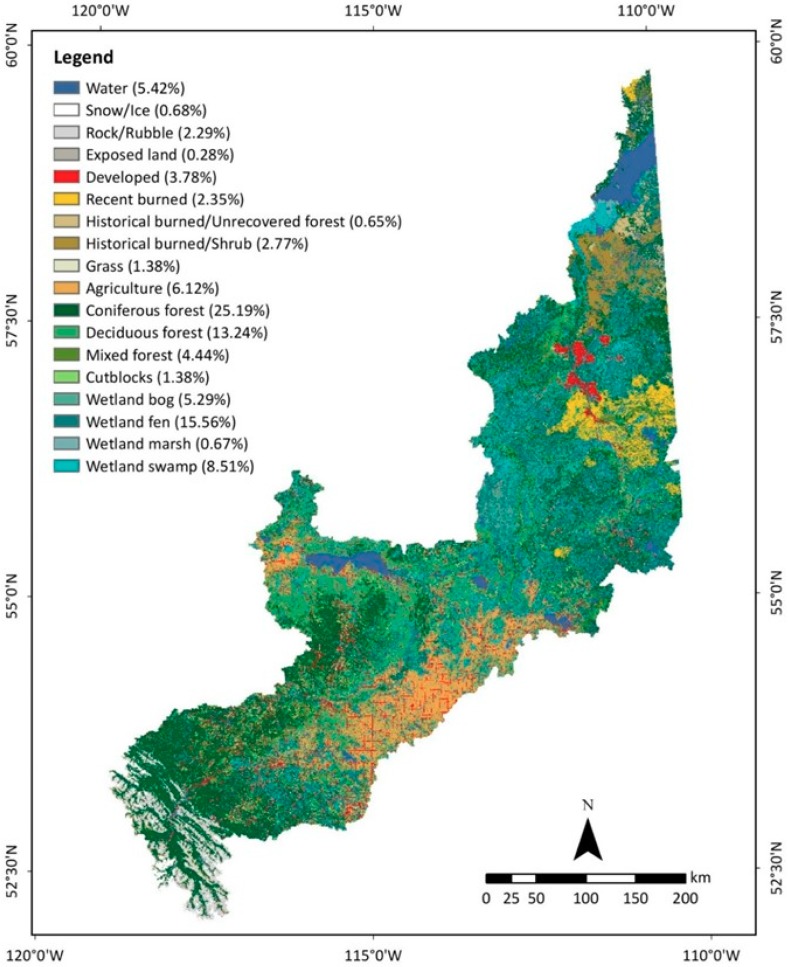
The first hydro-ecological map of the study area circa 2016 demonstrating 18 LULC classes.

**Figure 8:**
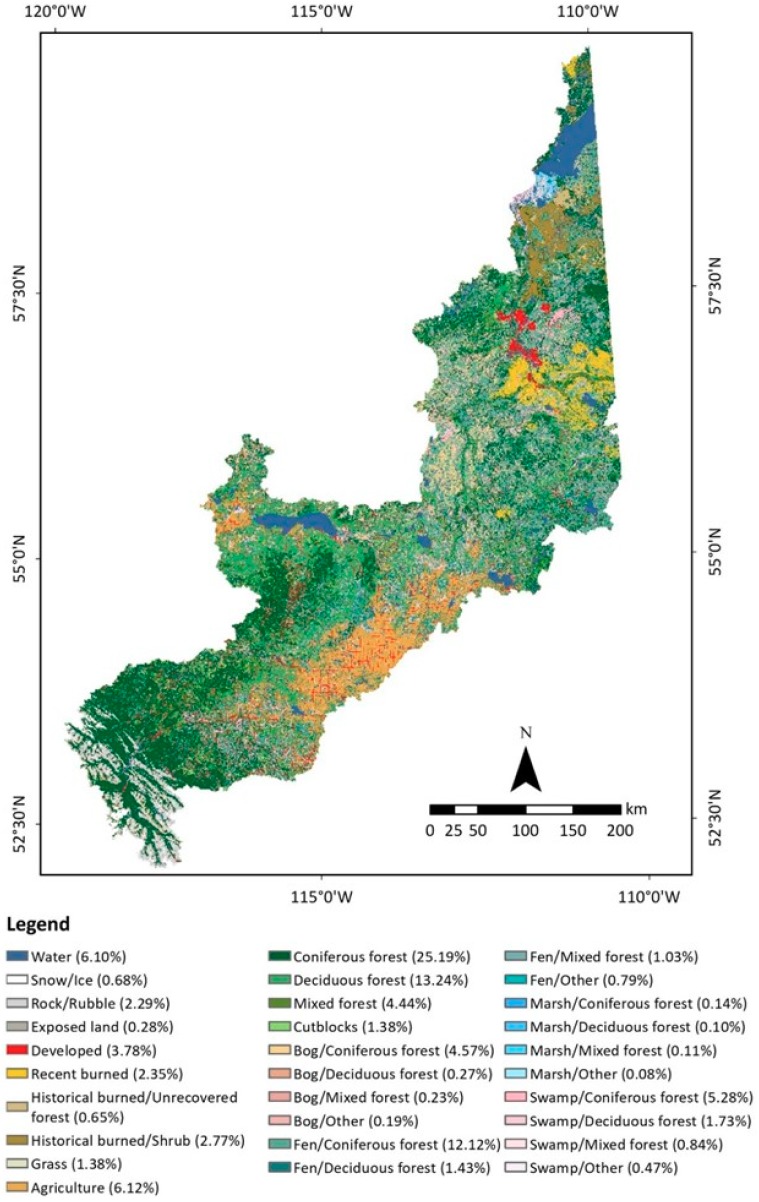
The second hydro-ecological map of the study area circa 2016 including 30 LULC classes.

According to the AMWI 2017 dataset, about 32% of the study area was covered by wetlands [40]. Therefore, integration of wetland information into the LULC map was important for hydro-ecological applications in the watershed. The results of the data fusion techniques are demonstrated using two hydro-ecological maps (Figure 7 and Figure 8), which could address the diverse needs of scientists. The first hydro-ecological map (Figure 7) revealed that about 30% of the watershed land was covered by four different types of wetlands: bog (5.29%), fen (15.56%), marsh (0.67%), and swamp (8.51%). Nearly 2% of the original wetlands were missed or lost in water, developed, or recent burned classes. This map also showed that the integration of the wetland information considerably reduced the coniferous forest, deciduous forest, mixed forest, and historical burned/shrub classes in the first hydro-ecological map compared to the final LULC map. By definition, wetland classes also included those vegetation classes.

The second hydro-ecological map (Figure 8) provides more detailed insight into different types of wetland compositions in the study area. The bogs and fens were heavily dominated by coniferous forest (4.57% and 12.12%, respectively), whereas swamps had a more balanced ratio (5:1) of coniferous and deciduous forests. Marsh lands were located at the edge of the water bodies, and they were mostly dominated by herbaceous plants. The presence of trees in the marshes was negligible. The identification of vegetation coverage within the wetlands could facilitate a detailed understanding of hydrological and hydrogeological processes within the watershed.

### 4.4. Potential Applications of the Hydro-Ecological Maps

The Athabasca River watershed management involves different user groups with diverse needs. Therefore, two hydro-ecological maps were prepared to meet the diverse needs of scientists, stakeholders, environmental managers, and regulators. In fact, these two maps (as shown in Figure 7 and Figure 8) are unique compared to other traditional LULC maps as they provide more detailed information about two specific categories, including different types of (i) burned class (approximately 6%) and (ii) wetland class (approximately 30%), in comparison to traditional LULC maps. It is widely acknowledged among scientists that wildfires and wetlands influence the hydro-ecological responses of a watershed to a great extent [51,52].

Under the burned class, three categories were included. Firstly, the recent burned category (2.35%) represented the infamous Fort McMurray fire (also known as Horse river fire), the costliest disaster in Canadian history [37]. It spread over 59,000 hectares of land, destroyed 2400 homes, and forced the evacuation of 88,000 people [52]. Moreover, this wildfire had a great toll on the local biodiversity of the study area due to the habitat loss of around 500 species in the burned boreal forest located there [52]. Secondly, the historical burned/unrecovered forest (0.67%) category depicted the areas that were burned by the wildfire events prior to 2011 fire season. Finally, the historical burned/shrub (3.39%) category showed the temporary changes in forest land cover due to recent wildfires which happened between 2011 and 2015. It is worthwhile to note that wildfire-induced burning may potentially impact the local-to-regional hydro-ecological processes in several ways, e.g., (i) by destroying local habitat and biodiversity [52], (ii) by releasing smoke, ash, and toxic gases into the atmosphere [53], (iii) by causing soil erosion from deforestation [54], and (iv) by contaminating soil and water from the nutrients resealed from dead plant and animal matter [54].

In the case of the wetland class, it was incorporated in two ways. The first involved the addition of four categories of wetlands including bog (5.29%), fen (15.56%), marsh (0.67%), and swamp (8.51%), as shown in Figure 7. The second involved the sub-categorization of each of the four categories of wetlands on the basis of vegetation coverage into further four types, i.e., bog/coniferous forest, bog/deciduous forest, bog/mixed forest, and bog/other for bog class (see Figure 8 for details). Wetlands occupy about one-third of the study area, and they would have a considerable influence on water balance (water storage, streamflow, and runoff), water quality, atmospheric carbon, nitrogen cycles, and wetland flora and fauna [55]. Also, the hydrologic regime could be affected by the land surface characteristics of the watershed, such as vegetation and soil properties. For example, forest and grassland could generate higher infiltration rates compared to cultivated field crops in the same climate conditions [56]. As the direct relationship between hydrological processes and LULC characteristics was proven in the literature, such as for overland flow [56], infiltration rates [57], and evapotranspiration losses [58], it would be essential to include the detailed characteristics of the land surface in eco-hydrological studies. For instance, Farjad et al. [56] investigated responses of hydrological processes to LULC changes in a watershed in southern Alberta. They demonstrated that changes in LULC could alter infiltration, baseflow, actual evapotranspiration, overland flow, and consequently the overall water balance of the watershed. Such studies extract land surface parameters, e.g., leaf area index and root depth for hydrological modeling from LULC maps. Human-induced modifications in the Athabasca watershed, such as deforestation and urbanization, could also alter the LULC characteristics of the watershed, resulting in changes in hydrological regime. Therefore, the generated LULC maps in this study with its eco-hydrological characteristics could play an important role in hydrology and ecological studies of the Athabasca watershed.

## 5. Conclusions

In this study, an integrated approach was followed to generate an LULC map of the Athabasca River watershed based on Landsat-8 OLI images and a time series of MODIS EVI images. The LULC map of the Athabasca River watershed circa 2016 provides comprehensive and current LULC information for the watershed. These updated LULC maps could serve as a vital source of information and data to further guide land-use decisions, including further urbanization and industrialization, in the watershed. Moreover, the requirements of different user groups were addressed by customizing the LULC maps for hydro-ecological applications, thereby giving users options to choose between two types of hydro-ecological map. Furthermore, an alternative approach of using multi-temporal MODIS/Terra 16-day composite EVI images was developed to delineate accurate vegetation coverage for the watershed. The use of multi-temporal MODIS EVI images compensated for the unavailability of multi-dated Landsat images required for withstanding the seasonal variability of vegetation classes. Nevertheless, the authors suggest that future studies should use multi-dated Landsat images, if possible, to generate an LULC map for any forest-dominated landscape. The results of the study also identified that the use of the ancillary data greatly improved the accuracy of the preliminary LULC map. The overall accuracy of the final LULC map circa 2016 was 74.95%, which is fairly reliable for a large watershed with heterogeneous landscapes. Based on this study experience, using ground-truth data in the validation process is recommended to avoid inconsistencies in the definitions of the reference data and the LULC map data. Ultimately, the use of the hydro-ecological maps for various environmental applications in the watershed was demonstrated. Ideally, the maps can be used by watershed planners and decision-makers as useful tools to formulate and implement different strategies and responses for sustainable watershed management in the study area.

## Figures and Tables

**Table 1:** Land-use and land-class (LULC) class coverage of the reference polygons and their totals in the study area.

LULC Class	Reference Area (km^2^)	Total Area (km^2^)	Percentage (%)
Water	182	9171	1.98
Snow/ice	11	1041	1.02
Rock/rubble	36	3472	1.02
Exposed land	10	980	1.01
Developed	118	5255	2.24
Recent burned	71	3541	1.99
Historical burned/shrub	66	5215	1.27
Grass	10	930	1.03
Agriculture	189	9786	1.93
Coniferous forest	746	73,531	1.01
Deciduous forest	460	26,910	1.71
Mixed forest	162	10,426	1.56
Total	2,060	150,256	1.37

**Table 2:** Confusion matrix for the 12 LULC classes of the final LULC map circa 2016 in the study area.

LULC Class	Water	Snow/ice	Rock/Rubble	Exposed Land	Developed	Recent Burned	Historical Burned/Shrub	Grass	Agriculture	Coniferous Forest	Deciduous Forest	Mixed Forest	Row Total	User’s Accuracy
Water	189,274	0	37	0	1550	24	896	0	154	5363	4965	2109	204,372	93%
Snow/ice	0	10,747	765	0	0	0	0	8	0	51	0	0	11,571	93%
Rock/rubble	4	1007	36,358	0	0	0	0	228	0	863	0	0	38,460	95%
Exposed land	207	0	0	10,214	133	0	160	4	483	7108	1199	1158	20,666	49%
Developed	187	0	0	0	123,597	1	131	0	2856	9018	13,074	4506	153,370	81%
Recent burned	0	0	0	0	110	71,907	1007	0	0	71	6	64	73,165	98%
Historical Burned/Shrub	3003	0	108	384	830	3829	67,715	72	0	33,087	3215	2494	114,737	59%
Grass	382	38	1808	37	42	0	81	10,034	0	5716	388	304	18,830	53%
Agriculture	46	0	0	0	606	0	0	0	200,195	521	8083	832	210,283	95%
Coniferous forest	6166	0	405	405	2542	307	2700	269	1572	673,284	131,912	81,212	900,774	75%
Deciduous forest	1562	0	0	0	1085	563	26	0	4851	50,555	278,410	45,358	382,410	73%
Mixed forest	993	0	0	0	331	1729	484	45	140	41,851	69,993	41,065	156,631	26%
Column total	201,824	11,792	39,481	11,040	130,826	78,360	73,200	10,660	210,251	827,488	511,245	179,102	2,285,269	
Producer’s accuracy	94%	91%	92%	93%	94%	92%	93%	94%	95%	81%	54%	23%		
						Overall accuracy = 74.95%								
						Cohen’s kappa = 0.68								
